# Association between stigma, depression and quality of life of people living with HIV/AIDS (PLHA) in South India – a community based cross sectional study

**DOI:** 10.1186/1471-2458-12-463

**Published:** 2012-06-21

**Authors:** Bimal Charles, Lakshmanan Jeyaseelan, Arvind Kumar Pandian, Asirvatham Edwin Sam, Mani Thenmozhi, Visalakshi Jayaseelan

**Affiliations:** 1 AIDS Prevention and Control Project, Voluntary Health Services, Adyar, Chennai, 600 113, India; 2 Department of Bio-Statistics, Christian Medical College and Hospital, Vellore, India; 3 USAID, New Delhi, India

## Abstract

**Background:**

India has around 2.27 million adults living with HIV/AIDS who face several challenges in the medical management of their disease. Stigma, discrimination and psychosocial issues are prevalent. The objective of the study was to determine the prevalence of severe stigma and to study the association between this, depression and the quality of life (QOL) of people living with HIV/AIDS (PLHA) in Tamil Nadu.

**Methods:**

This was a community based cross sectional study carried out in seven districts of Tamil Nadu, India, among 400 PLHA in the year 2009. The following scales were used for stigma, depression and quality of life, Berger scale, Major Depression Inventory (MDI) scale and the WHO BREF scale. Both Stigma and QOL were classified as none, moderate or severe/poor based on the tertile cut off values of the scale scores. Depression was classified as none, mild, moderate and severe. Logistic regression analyses were performed to study the risk factors.

**Results:**

Twenty seven per cent of PLHA had experienced severe forms of stigma. These were severe forms of personalized stigma (28.8%), negative self-image (30.3%), perceived public attitude (18.2%) and disclosure concerns (26%). PLHA experiencing severe depression were 12% and those experiencing poor quality of life were 34%. Poor QOL reported in the physical, psychological, social and environmental domains was 42.5%, 40%, 51.2% and 34% respectively. PLHA who had severe personalized stigma and negative self-image had 3.4 (1.6-7.0) and 2.1 (1.0-4.1) times higher risk of severe depression respectively (p < .001). PLHA who had severe depression had experienced 2.7(1.1-7.7) times significantly poorer QOL.

**Conclusions:**

Severe forms of stigma were equivalently prevalent among all the categories of PLHA. However, PLHA who had experienced severe depression had only developed poor QOL. A high level of social support was associated with a high level of QOL.

## Background

The provisional estimates in 2008 suggest an adult HIV prevalence of 0.29 per cent, amounting to 2.27 million people with HIV in India [1]. According to the National AIDS Control Organization’s (NACO) sentinel surveillance, the state of Tamil Nadu has the highest number of reported cases of AIDS. A large proportion of whom are increasingly accessing antiretroviral therapy [1]. People living with HIV/AIDS (PLHA) face several challenges in terms of the medical management of their disease. Alongside this are stigma, discrimination and psychosocial issues associated with HIV infection. HIV and AIDS related stigma is socially shared knowledge about the devalued status of people living with HIV that means treating someone as unimportant [2]. It is manifested in prejudice, discounting, discrediting and discrimination directed at people perceived to have HIV, along with the groups and communities to which they are associated. Literature revealed a higher level of stigma among PLHA in India [3-8]. A study showed that both PLHA and leprosy-affected people faced a substantial burden of internalized and perceived stigma, with the former reporting a significantly higher level [8]. Many instances of discriminatory behavior such as denial of hospital care, expulsion from the home and profession were also reported [9,10]. In Indian hospitals, stigma and discrimination manifested as health workers informing family members of HIV positive person’s status without consent, burning their bedding upon discharge, charging them more, and using gloves during all interactions [11]. A recent study found that enacted and internalized stigmas among PLHA were related to delays in seeking care [12]. The factors that contribute to HIV stigma and discrimination include, fear of transmission, fear of suffering and death and the burden of caring for PLHA [13]. Stigma thus, prevents HIV positive persons from disclosing their status to family, care providers, and sexual partners which also contributes to non-adherence to antiretroviral therapy [4,14].

Stigma has been shown to be associated with stress, depression and a lower quality of life (QOL) among PLHA [15-17]. Felt and internalized stigma has been associated with higher levels of depression [4,18,19]. A study indicated that enacted stigma, internalized stigma, and disclosure avoidance were all associated with symptoms of depression [20]. It is evident that depression and anxiety are commonly associated with HIV infection [21]. Depression is also reported as a well-recognized side effect of antiretroviral therapy (ART) in HIV infection [22]. Depression was reported to be present in 40% of HIV positive heterosexuals. The presence of pain, concurrent alcohol abuse, poor family relations and HIV positive status of the spouse were significantly associated with depression, anxiety and suicidal ideation [23]. Faster progression to AIDS was associated with more cumulative stressful life events, more cumulative depressive symptoms and less cumulative social support [24]. Also, chronic depression, stressful events and trauma are greatly associated with decreased CD4 T lymphocytes, increased viral load and mortality [25]. Apart from these, nervous system infection with HIV-1 can also produce a range of clinical disorders such as dementia, myelopathy and sensory neuropathies. However, these debilitating disorders generally do not develop until advanced stages of HIV infection [26,27].

HIV/AIDS stigma can severely compromise the quality of life (QOL) of people living with this condition by reducing access and quality of care. This affects adherence to therapy and thereby potentially increases the risk of transmission [28]. Some studies from India have reported the association between stigma and the Quality of Life (QOL). A clinic based study reported that internalizing of stigma had a significant negative correlation with QOL in the psychological domain and environmental domain [6]. PLHA experiencing higher stigma obtained lower scores in the psychological, environmental and Spirituality/Religious/Personal belief (SRPB) domains of the quality life [29]. Another clinic based study reported that each type of stigma was associated with each domain of QOL [5]. In India, the educational level of PLHA was significantly associated with the psychological domain of QOL; occupation and better family support of PLHA were significantly associated with the environmental domain of QOL [30]. Among PLHA, women were reported to have the poorest QOL but this was reported to have improved over time due to treatment [31]. However, none of the studies from India have explored the association between stigma, depression and QOL in PLHA. Interventions to facilitate HIV positive persons to effectively cope with HIV associated stigma are urgently required in India. Nevertheless, there is a dearth of information that specifies the need to develop such interventions. Based on the above review, we hypothesized that “severe stigma”, would be associated with severe depression and these both collectively or individually would correlate with QOL in PLHA. The objective of this study was to determine the prevalence of severe stigma and to study the association between stigma and depression on quality of life of PLHA in Tamil Nadu.

## Methods

### Sample size

The prevalence of severe stigma in PLHA was reported to be 23.6% (Manhart L, Kumar S, Mohanraj R and Jeyaseelan L, 2008[Unpublished Data]). Assuming a similar prevalence, 400 PLHA were studied. Based on 95% confidence interval, these 400 participants would give a precision of 4.16%.

### Sampling strategy

The study was conducted in seven Intensive Intervention Districts of the USAID supported AIDS Prevention and Control Project (APAC) in Tamil Nadu. The list of registered PLHA in these districts was obtained from the ART centres, PLHA network and NGOs of respective districts that served as a sampling frame. Probability proportionate to size (PPS) method was adopted to select the samples. The number of PLHA in each district (denominator) and the potential number to be selected from each district (denominator) are presented as follows. Kancheepuram (36/2599); Kanyakumari (21/1519); Karur (33/2397); Villupuram (41/2999); Trichy (176/12904); Thirunelveli (63/4632); Tuticorin (30/2209). Within each district a consecutive sample of PLHA who consented to participate in the study were recruited.

### Inclusion and exclusion criteria

PLHA aged between 18–60 years and who consented to participate were included in the study. PLHA who were too sick to answer the questions were not included in the study.

### Instruments

Data were collected by qualified and trained investigators using a structured interview schedule. The interview schedule contained questions on socio-demographic variables such as the age, sex (1.Male, 2.Female), religion (1. Hindu, 2.Muslim, 3.Christian), marital status (1.Unmarried, 2.Married, 3. Separated/Widowed), education (1.non-literate, 2.Primary, 3. High school and above), occupation (1.Casual labourer, 2.Agriculture, 3.Skilled and Semiskilled, 4.Sex work, 5.Others), personal income per month, alcohol intake (1.every day or 2-3times a week, 2.At least once a week, 3. Did not drink in the last 4 weeks, 4.Never), and current status of relationship with whom the study participant is living with (1.alone, 2.with parents, 3. with parents and children, 4.others). The instruments were translated from English into the local language (Tamil) and back translated to English.

### Stigma

Stigma was measured using the Berger scale [32]. This 40-item four point scale groups stigma into the following 4 categories, personalized stigma (self-stigma); perceived public attitude (concern with public attitude about people with HIV); disclosure concerns and negative self-image (internalized negative self-image). The scores are scaled in the positive direction (higher the score higher the stigma). Personalized stigma had items that assessed whether PLHA had experienced rejection, loss of employment, and discrimination and therefore stopped socializing. Negative self-image had items that assessed the fear of being stigmatized, concerns about people’s reactions towards people with HIV, individual beliefs and feeling guilty. Public attitude had items that indicated public reactions towards HIV; for instance, the public view that a HIV person is dirty and disgusting, and other attitudes of discrimination that included normal people treating PLHA like outcasts. Disclosure concerns had items which assessed anxiety and fear of disclosing their HIV status. The scale was pilot tested for its reliability in this cultural setting. The internal reliability (Cronbach’s Alpha) was 0.79. In this study, each domain and the overall stigma scores were categorized into three categories such as no or mild, moderate and severe stigma using the 33^rd^ and 66^th^ percentile cut off values from the distribution of scores. This categorization was exclusively done for this study.

### Depression

Depression was measured using the Major Depression Inventory (MDI), which is a diagnostic tool [33,34]. This self-rating scale was developed by the World Health Organization and consists of 10 items measuring the severity of depressive states. The items are rated on a 6 point Likert scale ranging from 0 (symptom has not been present at all) to 5 (symptom has been present all the time) and individuals are categorized as normal, mild, moderate or severe. The criteria for classification are provided in the above reference. In the analyses, depression was classified as severe and others. A total MDI score of 30 or above was considered as severe depression. For reliability, test retest reliability over time (with a break of 2 weeks) and across items was done with 20 PLHA. The investigators were trained by Clinical Psychologists.

### Quality of life

Quality of life was measured using the WHO BREF scale with 26 items [35]. This instrument has 4 domains, which are physical, psychological, social and environmental. The physical domain has 7 items which included questions about the presence of pain and discomfort, dependence on substances or treatments, energy and fatigue, mobility, sleep and rest; activities of daily living; perceived working capacity. The psychological domain has areas such as negative self-concept, cognitive functions, body image and spirituality. The social domain has 3 questions which are about social contacts, family support, ability to look after family and sexual activity. The environment domain has 8 items, which are freedom, quality of home environment, physical safety and security and financial status, involvement in recreational activity, health and social welfare, health and social care and quality and accessibility. The domain scores were scaled in a positive direction, implying that higher the score, higher the quality of life. A total score for each domain and an overall QOL score were calculated. The distributions of the domain and over all scores were divided into three groups such as, poor, moderate and good quality of life based on the 33^rd^ and 66^th^ percentile cut off values. The instruments were pilot tested with 20 participants, who were not included in the main study. Each interview took nearly 40 to 50 minutes.

### Data analyses

SPSS 16.0 was used for analyses. In addition to descriptive analyses, bivariate analyses were carried out to determine the association between the socio demographic variables, HIV program related variables and outcome variables that are Stigma, Depression and QOL. The variables which were significant at p < .40 were considered as potential risk factors for multivariate analyses. However, the different domains of stigma and access to ART were included in the model irrespective of their significance. Enter method was used to obtain odds ratio and 95%CI. Hosmer and Lemeshow chi-square test were used to assess the Goodness of fit of the model. Cox and Snell R^2^ were used.

### Reliability of instruments

In the Berger scale, the overall internal consistency (Cronbach’s alpha) for the entire 40-item scale was 0.79. The Cronbach’s alpha for self, public attitude, disclosure and negative stigma was .76, .79, .62 and .85 respectively. The test and retest reliability for the overall 40 items was 0.89. In QOL, the overall internal consistency was .81. This was .75, .82, .85, and .79 for physical, psychological, social and environmental domains. The test retest reliability was .83.

### Adherence to ethical standards

The study protocol conformed to the Declaration of Helsinki and Indian Council of Medical Research (ICMR) ethical guidelines. The ethical approval was obtained from Institutional Review Board of Christian Medical College and Hospital, Vellore, India.

## Results

### Socio demographic profile, stigma, depression and quality of life

Table 1 and Table 2 present the socio demographic characteristics, stigma, depression and QOL of PLHA by gender. The study included 188 (47%) males and 212 (53%) females. Men and women aged ≤ 30 years were 29.3% and 21.7% respectively. A higher proportion of women(61.8%) were in the age group of 31–40 years as compared to only 48.4% of men in the same age group. 22.3% of men and 16.5% of women were from ≥41 year’s age group. 11.2% of men were non-literate, while it was 17.5% among women (p < .01). Nearly one third of men and 5.7% of women were unmarried. Nearly half the women were separated or divorced as compared to 13.8% of men (p < .001). Nearly one fourth of men were living with their parents, compared to only 6.6% of women. Significantly more women (71.2%) were living with their spouses and/or children as compared to men (47.3%, p < .001). There were also significantly more men employed, earning more income and consuming alcohol as compared to women (p < .001). 71.8% of participants received ART care and there was no significant difference based on gender. Nearly one fourth of the participants received high social support from relatives and friends, while 41% and 34.7% received low and moderate levels of social support respectively.

**Table 1:** Distribution of Socio demographic variables by gender

**Variables**	**Male n=188**		**Female n=212**		**Total**		**P-Value**
	**N**	**%**	**N**	**%**	**N**	**%**	
**Age**							
< = 30	55	29.3	46	21.7	101	25.2	0.027
31–40	91	48.4	131	61.8	222	55.5	
> = 41	42	22.3	35	16.5	77	19.2	
**Education**							
Non- literate	21	11.2	37	17.5	58	14.5	0.008
Primary/secondary	23	12.2	43	20.3	66	16.5	
High School & above	144	76.6	132	62.3	276	69.0	
**Marital Status**							
Unmarried	64	34.0	12	5.7	76	19.0	0.000
Married	98	52.1	87	41.0	185	46.2	
Sep/Widowed	26	13.8	113	53.3	139	34.8	
**Living with Whom**							
Alone	34	18.1	18	8.5	52	13.0	0.000
Parents	48	25.5	14	6.6	62	15.5	
With Spouse & or children	89	47.3	151	71.2	240	60.0	
Others	17	9.0	29	13.7	46	11.5	
**Employment**							
Employed	171	91.0	155	73.1	326	81.5	0.000
Unemployed/Housewife/retired	17	9.0	57	26.9	74	18.5	
**Occupation**							
Casual Labourer	50	26.6	36	17.0	86	21.5	0.000
Agriculture	20	10.6	14	6.6	34	8.5	
Skilled and semiskilled	55	29.3	22	10.4	77	19.2	
Sex work	6	3.2	34	16.0	40	10.0	
Others	57	30.3	106	50.0	163	40.8	
**Income**							
< = 2000	40	23.4	66	42.6	106	32.5	0.001
2000–3000	78	45.6	60	38.7	138	42.3	
3001 +	53	31.0	29	18.7	82	25.2	
**Alcohol**							
Every day or 2-3 times in a week	18	9.6	11	5.2	29	7.2	0.000
At least once in week	22	11.7	7	3.3	29	7.2	
Did not drink in the last 4 week	51	27.1	19	9.0	70	17.5	
Never	97	51.6	175	82.5	272	68.0	
**Access to Care: ART centres**							
Yes	138	73.4	149	70.3	287	71.8	0.489
No	50	26.6	63	29.7	113	28.2	
**Drop in centres**							
Yes	52	27.7	52	24.5	104	26.0	0.476
No	136	72.3	160	75.5	296	74.0	
**Comprehensive continuum of HIV care**							
Yes	46	24.5	56	26.4	102	25.5	0.656
No	142	75.5	156	73.6	298	74.5	

**Table 2:** Distribution of outcome variables by gender

**Variables**	**Male**		**Female**		**Total**		**P-Value**
	**N**	**%**	**N**	**%**	**N**	**%**	
**Personalized Stigma**							
None/Moderate	141	75.0	144	67.9	285	71.2	0.119
Severe	47	25.0	68	32.1	115	28.8	
**Negative Self image**							
None/Moderate	128	68.1	150	71.1	278	69.7	0.515
Severe	60	31.9	61	28.9	121	30.3	
**Perceived Public Attitude**							
None/Moderate	143	76.1	184	86.8	327	81.8	0.006
Severe	45	23.9	28	13.2	73	18.2	
**Disclosure Concerns**							
None/Moderate	137	72.9	159	75.0	296	74.0	0.628
Severe	51	27.1	53	25.0	104	26.0	
**Overall Stigma**							
None/Moderate	143	76.1	148	70.1	291	72.9	0.184
Severe	45	23.9	63	29.9	108	27.1	
**Depression**							
Severe	22	11.7	26	12.3	48	12.0	0.863
Others	166	88.3	186	87.7	352	88.0	
***Quality of Life***							
**Physical Domain**							
Poor	83	44.1	87	41.0	170	42.5	0.530
Others	105	55.9	125	59.0	230	57.5	
**Psychological Domain**							
Poor	68	36.2	92	43.4	160	40.0	0.141
Others	120	63.8	120	56.6	240	60.0	
**Social Domain**							
Poor	89	47.3	116	54.7	205	51.2	0.141
Others	99	52.7	96	45.3	195	48.8	
**Environment**							
Poor	65	34.6	71	33.5	136	34.0	0.819
Others	123	65.4	141	66.5	264	66.0	
**Overall Quality of Life**							
Poor	67	35.6	69	32.5	136	34.0	0.515
Others	121	64.4	143	67.5	264	66.0	
**Social Support**							
Low	80	42.6	84	39.6	164	41.0	0.530
Moderate	60	31.9	79	37.3	139	34.8	
High	48	25.5	49	23.1	97	24.2	
**Are you a member of any association**							
Yes	78	41.5	106	50.0	184	46.0	0.088
No	110	58.5	106	50.0	216	54.0	

The prevalence of severe stigma was 27.1% (22.8-31.5) and the prevalence of severe personalized stigma, negative self-image, perceived public attitude and disclosure concerns were 28.8% (24.3-33.2), 30.3% (25.7-34.8), 18.2% (14.4-21.9) and 26% (21.7 -30.2) respectively. The prevalence of severe depression was 12% (8.8-15.2). The prevalence of overall poor QOL was 34% (29.3-30.6). Poor QOL under physical, psychological, social and environmental domains was 42.5% (37.5-47.3), 40% (35.1-44.8), 51.2% (46.3-56.1) and 34% (29.3-38.6) respectively. The bivariate analysis did not indicate any significant difference by gender in stigma, depression and QOL except public attitude stigma which indicated a significant difference (p < .01).

### Stigma and quality of life

The distribution of types of stigma according to different domains of QOL is presented in Table 3. The PLHA who had severe self-stigma were 1.4 (1.1 – 1.8) times significantly more likely to have poor QOL in the environmental domain (p < .05). Similarly, PLHA who had severe disclosure concerns were 1.8(1.4 – 2.4) times significantly more likely to have poor QOL in the environmental domain (p < .001). Otherwise, there was no association between types of stigma and domains of QOL. However, severe over all stigma was associated with poor QOL in the social domain (p < .01).

**Table 3:** Bivariate Analysis of Stigma, Depression and Quality of life

**Types of stigma & Depression**	**N**	**Quality of Life**																			
		**Physical Domain**					**Psychological Domain**					**Social Domain**					**Environment Domain**				
		**Poor**		**RR**	**95% CI**	**P value**	**Poor**		**RR**	**95% CI**	**P value**	**Poor**		**RR**	**95% CI**	**P value**	**Poor**		**RR**	**95% CI**	**P value**
		**n**	**%**				**n**	**%**				**n**	**%**				**n**	**%**			
**Personalized**																					
Severe	115	45	39.1	0.89	0.69–1.16	0.386	46	40.0	1.00	0.77–1.30	1.000	65	56.5	1.15	0.94–1.41	0.180	49	42.6	1.40	1.06–1.84	0.021
Others	285	125	43.9				114	40.0				140	49.1				87	30.5			
**Negative**																					
Severe	121	44	36.4	0.81	0.62–1.06	0.110	40	33.1	0.77	0.58–1.03	0.068	65	53.7	1.07	0.88–1.32	0.494	36	29.8	0.83	0.60–1.13	0.228
Others	278	125	45.0				119	42.8				139	50.0				100	36.0			
**Public Attitude**																					
Severe	73	34	46.6	1.12	0.85–1.48	0.435	33	45.2	1.16	0.87–1.55	0.315	35	47.9	0.92	0.71–1.20	0.532	23	31.5	0.91	0.63–1.32	0.618
Others	327	136	41.6				127	38.8				170	52.0				113	34.6			
**Disclosure**																					
Severe	104	52	50.0	1.25	0.99–1.59	0.072	49	47.1	1.26	0.98–1.62	0.085	55	52.9	1.04	0.84–1.29	0.698	53	51.0	1.82	1.40–2.36	< 0.001
Others	296	118	39.9				111	37.5				150	50.7				83	28.0			
**Overall**																					
Severe	108	41	38.0	0.86	0.66–1.13	0.279	39	36.1	0.88	0.66–1.19	0.352	67	62.0	1.32	1.09–1.60	0.008	39	36.1	1.08	0.80–1.46	0.603
Others	291	128	44.0				120	41.2				137	47.1				97	33.3			
**Depression**																					
Severe	48	19	39.6	0.92	0.64–1.34	0.663	25	52.1	1.36	1.0–1.84	0.069	28	58.3	1.16	0.89–1.51	0.295	23	47.9	1.49	1.07–2.08	0.030
Others	352	151	42.9				135	38.4				177	50.3				113	32.1			

### Depression and quality of life

The distribution of depression by different domains of QOL is presented in Table 3. PLHA who experienced severe depression were 1.4 (1.0-1.8; p = .07) and 1.5 (1.1 – 2.1; p < .05) times more likely to have experienced poor psychological and environmental QOL respectively.

### Multivariate analyses of overall stigma, depression and overall quality of life

The results of logistic regression analyses of overall stigma, depression and overall QOL are presented in Table 4. The non-literates and those who studied up to primary level were significantly more likely to experience severe stigma as compared to those who studied up to high school and above (p < .01). The PLHA who were accessing ART services were significantly more likely to experience severe stigma (OR = 2.2; 95% CI: 1.2-4.1, p < .01).

**Table 4:** Multivariate Analysis of Socio-Demographic variables and stigma with Depression and Quality of Life

**Variables**	**Stigma**			**Depression**			**Quality of Life**		
	**OR**	**95% CI**	**P Value**	**OR**	**95% CI**	**P Value**	**OR**	**95% CI**	**P Value**
**Age**									
< = 30				1.21	0.45–3.27	0.710			
31–40				0.63	0.27–1.50	0.299			
> = 41				1.00					
**Gender**									
Male	0.82	0.45–1.50	0.526						
Female	1.00								
**Education**									
Non- literate	2.33	1.20–4.51	0.012				2.01	0.92–4.39	0.080
Primary/secondary	2.25	1.21–4.20	0.011				1.33	0.60–2.95	0.477
High School & above	1.00						1.00		
**Marital Status**									
Unmarried	1.00			1.00			1.00		
Married	1.34	0.66–2.74	0.417	5.79	1.81–18.54	0.003	0.72	0.33–1.59	0.419
Sep/Widowed/Divorce	1.30	0.58–2.90	0.529	1.98	0.56–6.93	0.287	0.85	0.39–1.87	0.685
**Occupation**									
Casual Labourer	1.48	0.77–2.82	0.237				2.99	1.37–6.51	0.006
Agriculture	1.99	0.82–4.84	0.125				1.36	0.47–3.95	0.572
Skilled and semiskilled	1.57	0.79–3.18	0.207				1.52	0.64–3.66	0.344
Sex work	0.74	0.27–2.01	0.555				2.66	0.88–8.03	0.083
Others	1.00						1.00		
**Income**									
< = 2000							2.53	1.02–6.23	0.044
2000–3000							1.67	0.77–3.65	0.194
3001 +							1.00		
**Alcohol**									
Every day or 2-3 times in a week	0.40	0.13–1.24	0.112						
At least once in week	0.74	0.28–1.93	0.535						
Did not drink in the last 4 week	0.51	0.24–1.06	0.072						
Never	1.00								
**Access to Care: ART centers**									
Yes	2.25	1.24–4.10	0.008	0.94	0.44–2.03	0.884	2.17	1.13–4.15	0.019
No	1.00			1.00			1.00		
**Drop in Centers**									
Yes	1.47	0.83–2.61	0.182	0.59	0.26–1.38	0.225	0.90	0.46–1.77	0.760
No	1.00			1.00			1.00		
**Comprehensive continuum of HIV care**									
Yes	0.81	0.44–1.48	0.495						
No	1.00								
**Disclosure concerns**									
No/Moderate				1.00			1.00		
Severe				0.59	0.26–1.35	0.212	1.49	0.80–2.75	0.207
**Self-Stigma**									
No/Moderate				1.00			1.00		
Severe				3.42	1.67–6.99	0.001	0.73	0.36–1.47	0.381
**Negative stigma**									
No/Moderate				1.00			1.00		
Severe				2.07	1.02–4.18	0.042	0.53	0.26–1.07	0.075
**Public Attitude**									
No/Moderate				1.00			1.00		
Severe				1.76	0.76–4.07	0.185	0.79	0.37–1.70	0.559
**Depression**									
Severe							2.73	1.11–6.71	0.028
Others							1.00		
**Member of any Association**									
Yes	0.77	0.46–1.28	0.311	1.00			1.81	0.96–3.41	0.067
No	1.00			4.10	1.90–9.10	0.001	1.00		
**Social Support**									
Low	1.92	0.94–3.90	0.071	1.81	0.73–4.49	0.199	12.12	4.30–34.11	0.001
Moderate	1.77	0.85–3.71	0.127	0.78	0.28–2.20	0.645	3.30	1.13–9.63	0.029
High	1.00			1.00			1.00		

Married PLHA were 5.7 (1.8-18.5) times more likely to have severe depression as compared to single PLHA (p < .01). Those who experienced severe personalized and negative stigma were 3.4 (1.6-6.9) and 2.1 (1.0-4.1) times respectively more likely to have severe depression (p < .05). PLHA who were not members of any association were 4.1 (1.8-9.0) times significantly more likely to have experienced severe depression (<.001).

The multivariate analysis indicated poor QOL among casual labourers, lower income group (<=2000 INR per month) and those who received less or moderate social support (p < .05). It also found a significant association between severe depression and poor QOL (OR = 2.7; 1.1-6.7, p < .05). A significant association was found between availing ART services and poor QOL (OR = 2.1; 1.1-4, 1, p < .05).

## Discussion

This was a community based study among PLHA in South India that determined the prevalence of severe stigma and the association between stigma and depression on quality of life. Some other studies carried out in southern India were primarily hospital based and did not focus on depression and quality of life [5,6].

Despite huge efforts in addressing stigma and discrimination, 27.1% of PLHA had experienced severe forms of overall stigma and 28.8%, 30.3%, 18.2% and 26% of them continue to experience severe forms of personalized stigma, negative self-image, public attitude stigma and disclosure concerns respectively. Some other studies also reported a higher level of stigma [5,6], especially “negative self-image” was reported to be common but reports of “self-stigma” were found to be low [3-5]. Another study revealed that actual stigma experienced among those infected with HIV was much less (26%) as compared to the fear of being stigmatized or perceived stigma (97%) [6]. Overall stigma was reported higher among non-literates and those who accessed ART services in this study. A study reported that accessing ART services would protect against stigma [12]. Preliminary data from research in rural Haiti suggest that the introduction of quality HIV care can lead to a rapid reduction in stigma, resulting in increased rate of seeking HIV services [36]. On the contrary, this study found that accessing ART services was associated with severe stigma and poor QOL which could be attributed to the discrimination shown against non-literate and economically poor PLHA at the facilities [10,18,37,38].

The prevalence of severe depression was found to be 12% among PLHA. Several studies reported a high prevalence of psychiatric disorders including depressive disorders among PLHA [39,40]. Unlike other studies conducted in low income countries, this study found that married PLHA were more likely to have depression and the potential reasons could be the responsibility to take care of the children and family and fear of disclosing the status to the family members due to concerns of losing social and economic support [41-43]. It was also found that being a member of any association was associated with less risk for depression as indicated in some other studies [44,45]. This study revealed that those who experienced self and negative stigma had a significantly higher prevalence of severe depression which is in corroboration with studies carried out in India and South Africa [4,15,17,23]. It was reported that enacted stigma, internalized stigma, and disclosure avoidance were all associated with depression symptoms [4,20]. But, a study carried out in South India among women HIV positives did not find any association between stigma and depression [46]. Unlike other studies, no association was found between disclosure concerns and self or negative stigma in this study. Similarly there was no significant association between disclosure concerns and depression. This could be due to the fact that the participants were from known PLHA networks and their status was not a hidden factor.

Given the availability of current prophylactic and therapeutic strategies for PLHA, quality of life (QOL) has emerged as a significant medical outcome measure. This study reported poor QOL among 34% of participants. QOL was markedly affected in Social domain (poor QOL 51.2%) as compared to other domains such as physical (42.5%), psychological (40%) and environmental (34%). Some other studies also reported poor QOL in different domains [47,48]. In this study, PLHA who were found to have personalized stigma and disclosure concerns had poor QOL in the environment domain only. The other domains of QOL did not have any associations with different types of stigma. But, stigma was found to have a significant negative correlation with QOL in some other studies [6,28,29,49]. On the contrary, a study showed that respondents who reported of actual stigma (33%) had significantly good QOL in their physical domain (49%), psychological domain (48%) and environmental domain (44%) [5].

According to this study, PLHA who were generally poor (casual labourers, earning < =Rs2000 per month), severely depressed and receiving lower social support had significantly poor QOL. These findings were similar to other clinic based studies [5,6,29,30]. Many studies have mentioned about the association between depression and QOL [40,50,51]. Also, higher social support was associated with lower depression and higher QOL, which is in corroboration with other studies [44,45]. In this study, PLHA who accessed ART were found to have poor QOL. This indicates that accessing ART services alone may not necessarily improve QOL, which suggests the need for strengthening interventions with more emphasis on emotional and psychological support [52]. The study findings suggest a need to strengthen social support network and programs for PHLA so as to reduce stigma.

This study found an association between different forms (personalized and negative) of stigma and severe depression. Severe depression was also associated with poor QOL. Though the study design did not allow us to prove casual association between stigma and poor quality of life, PLHA who experienced severe depression due to severe stigma were probably the PLHA who experienced poor quality of life. This calls for intensive social and psychological interventions among them.

As this study is cross sectional, it is difficult to prove causal relationships. The sampling procedure was dependent on the members of various PLHA networks. The proportion of PLHA who are not members of these networks, and those who have not disclosed their HIV status so far is unknown. In addition, PLHA aged between 18–60 years, who provided informed consent and were not too sick to answer the questions alone were included in the study. Thus our findings may not represent the entire PLHA in the State. As in many studies, we used the Berger HIV stigma scale, but the scale was not modified for Indian conditions [5,6,14].

## Conclusions

In summary, this community based study found a prevalence of severe stigma of 27%. Severe depression and poor overall QOL were 12% and 34% respectively. Personalized and negative stigma were significantly associated with severe depression. A high level of social support was associated with a high level of QOL. Accessing ART service was significantly associated with severe stigma and poor QOL. It may be concluded that, ensuring high quality comprehensive services at the ART centres and a high level of social support for the PLHA are vital and would lead to a decrease in depression and an increase in QOL.

## Competing interests

The authors declare that they have no competing interests.

## Authors’ contributions

BC designed and coordinated the study, performed analyses and wrote the manuscript. LJ, AKP and AES contributed to the study design, data analysis, and interpretation of results and preparation of manuscript. MT and VJ contributed to data analysis and results interpretation. All authors read and approved the final manuscript.

## Pre-publication history

The pre-publication history for this paper can be accessed here:

http://www.biomedcentral.com/1471-2458/12/463/prepub
